# Vesical Calculi in the Female—Report of a Case

**Published:** 1895-04

**Authors:** Frederick J. Mann

**Affiliations:** Watertown, N. Y.; Formerly of Resident Staff Buffalo General Hospital


### VESICAL CALCULI IN THE FEMALE—REPORT OF A CASE.

By FREDERICK J. MANN, Ph. B., M. D., Watertown, N. Y.
Formerly of Resident Staff Buffalo General Hospital.

I AM enabled to report the following case through the courtesy of Dr. M. D. Mann and Dr. Chas. Cary, who were attending at the hospital when the case occurred.

Mrs. H., age 72, German, widow, was brought to the general hospital in the ambulance in a semi-conscious condition, probably due to a slight cerebral hemorrhage, from which she recovered in two or three days. Has had five children who would now be from 41 to 49 years of age. She has lived alone for the past fourteen years, leading a hermit life, being considered "queer," perhaps harmlessly insane, by her family. She gave a very incomplete medical history of herself.

On admission, patient was found to have a tumor protruding from her vulva, measuring about eight inches in diameter, which so projected between her legs as to make it impossible for her to lie on her back. It was somewhat ulcerated from contact with her thighs. This had existed for thirty years, being at first small and for the last few years increasing rapidly in size. Examination showed it to be a prolapsus of the uterus and vagina. Under an anesthetic it was easily reduced by taxis; gurgling, showing it to be an enterocele. After reduction a tampon was placed to keep the uterus in place and the patient put to bed.

Two days later twelve vesical calculi were passed during micturition and three hours after this, some of the tampons having meanwhile been removed, eleven more calculi were passed, some of these later the nurse having to pick out from the meatus urinarius. Again, the next day, three more were passed, making a total of twenty-four. A careful watch of the urine was kept during the following three weeks that the patient remained in the hospital, but no more calculi were found.

The calculi were facetted, polyhedral in shape, practically uniform as to size, being about one-fourth inch to three-eighths inch in diameter and composed of uric acid with a phosphatic crust. The urine, while in the hospital, showed presence of neutral, tripple and amorphous phosphates and was slightly alkaline, otherwise normal. On account of the mental state of the patient no further reliable information as to previous condition could be obtained. The case is interesting as showing the result of a possible twisting and partial compression of the urethra by a severe prolapse, thereby giving a tendency to the formation and retention of calculi, just as occurs in the male. The uterus remained in its proper place during the three weeks the patient stayed in the hospital, the tampons having been removed. She then left the hospital and was lost sight of.

